# Computational and Mathematical Modeling of Tumor Kinetics and Response to Radiation and Chemotherapy

**DOI:** 10.1155/2012/702675

**Published:** 2012-12-20

**Authors:** Eva Bezak, Loredana Marcu, Scott Penfold

**Affiliations:** ^1^Department of Medical Physics Royal Adelaide Hospital, Adelaide, SA 5000, Australia; ^2^School of Chemistry and Physics, The University of Adelaide, Adelaide, SA 5005, Australia; ^3^Faculty of Science, University of Oradea, Universitatii str., Oradea, Romania

The AACR Cancer Progress Report (2011) shows that in the USA from 1990 to 2007, death rates from all cancers dropped to 22% in men and 14% in women. More than 68% of adults live five years or more after diagnosis, up from 50% in 1975. For all paediatric cancers, the five-year survival rate is 80%, compared with 52% in 1975. However, the poor survival rates from most deadly cancers, pancreatic, ovarian and glioblastoma multiforme (GBM), have not changed to this date.

Despite many technological and clinical advances, cancer death rates are still high. Patient death arises from the failure to detect primary tumors before they spread their micrometastases around the body, as in pancreatic and ovarian cancers, the inability to control primary tumors, as in GBM, or the inability to eliminate micrometastases before they form new lesions. Disseminated disease is the primary cause of cancer death.

On top of this is the rapidly developing cost of the management of cancer. Cancer causes the highest economic loss of all of the 15 leading causes of death worldwide. The World Health Organisation (WHO) notes that the economic toll from cancer is nearly 20% higher than heart disease, the second leading cause of economic loss ($895 billion and $753 billion, resp.). Health budgets in developed countries are continually expanding and constrained by limited resources. But cost/benefit analyses to determine whether the increased cost of treatment achieves improved outcomes remain unknown.

Approaches to understanding the causes and treatment of cancer are numerous, involving chemistry, physics, biology, genetics, medicine, pharmacology, and experiments based on *in vivo* or *in vitro* setups, using animal models and/or human trials. Among the plethora of experimental and research activities, mathematical models of cancer growth, behaviour, and response of tumors to agents like chemotherapy and radiotherapy have been the subject of intensive research and development since the middle of the last century. Just like all other cancer research, cancer modeling requires a multidisciplinary approach, involving physicists, mathematicians, molecular biologists, medical specialists, and many others. At present, the rapid growth of computational power allows us to build complex mathematical models that can investigate different aspects of the disease and can be used to investigate the role of complex tumor behaviour and its response to various therapeutic protocols.

In general, modeling methods can be divided into analytical methods and stochastic methods. Analytical methods are based on deterministic equations in order to model evolution of a biological system. A set of differential equations with multiple parameters is often used to model complex tumor kinetics under various conditions. On the other hand, stochastic methods use random number generators and probability distributions to simulate evolution of the system from a set of initial conditions and parameter values (also known as *in silico* models). These methods are particularly useful as they can take into account the high degree of complexity and multifaceted nature of cellular proliferation, communication, and interactions with reagents that cannot be easily described by a set of equations. 

Pros for modeling in cancer research are as follows.

- Modeling significantly reduces the time needed for an outcome as compared to *in vivo* techniques; it also reduces the costs involved in the research and, very importantly, reduces the risks, which, for example, in a clinical trial are unavoidable.
- Modeling allows the easy investigation of the “what if?” scenarios; that is, models have predictive power.
- Modeling allows for the quantitative assessment of qualitative processes.
- Because of the low costs involved, modeling allows researchers with modest infrastructure to contribute with valuable ideas to the field.
- Modeling enables to quantify and interpret experimental results, including data obtained from clinical trials.

Cons are as follows.

- It is very difficult, if not impossible, to model the entirety of a biological process.
- There is always a compromise in a modeling process as only a certain number of known biological processes/parameters can be accounted for within the model. This known compromise exists on top of the unknown compromise represented by the obvious omission of the unknown processes that happen in the modeled biological system.
- The results of an *in silico* model should be considered clinically only after the credibility of the model has been validated by several *in vivo* results.

Models can also allow for individualization of treatments. It is therefore possible that in near future a patient can receive individualized treatment based on their specific biological/genetic parameters rather than undergoing a treatment protocol that is based on population averages.

In this special issue we will explore the topic of computational and mathematical modeling of tumor kinetics and response to radiation and chemotherapy. The papers in this issue have been contributed by eminent researchers and research groups in the area of cancer modeling, radiation biology modeling, microdosimetry, and many others. Several papers offer comprehensive reviews of current status of cancer modeling in specific areas (e.g., hypoxia, glioblastoma multiforme models, etc.). We trust that the readers will find this issue both useful and practical as well as a good source of reference material and a stimulating read.

*Eva Bezak* *Loredana Marcu* *Scott Penfold*

